# Evaluation of Aggregometery Parameters and Efficacy of Plavix versus Clopidex in Patients Suffering from Ischemic Heart Disease: A Randomized Double Blind Clinical Trial

**DOI:** 10.5812/ircmj.15277

**Published:** 2014-02-07

**Authors:** Majid Shohrati, Maryam Moshkani, Bahram Pishgoo, Minoo Ahmadinejad, Nastaran Najafian, Bita Najafian, Davoud Kazemisaleh

**Affiliations:** 1 Chemical Injuries Research Center, Baqiyatallah University of Medical Sciences, Tehran, IR Iran; 2 Atherosclerosis Research Center, Baqiyatallah University of Medical Sciences, Tehran, IR Iran; 3 Department of Biochemistry, Payam Nour University, Tehran, IR Iran; 4 Department of Pediatrics, Baqiyatallah University of Medical Sciences, Tehran, IR Iran

**Keywords:** Diphosphates, Clopidogrel, Heart Disease

## Abstract

**Background:**

Ischemic heart disease is the leading cause of death in most societies. In a pathophysiologic point of view, it chiefly results from the formation of thrombus in coronary arteries which could not be only prevented by aspirin. Many of clinical trials have shown the long-term benefits of antiplatelet drugs in reducing the risk of thrombotic accidents.

**Objectives:**

Clopidogrel is a thienopyridine derivative used to prevent platelets from adhering together by direct inhibition of Adenosine diphosphate (ADP), the major factor behind platelets aggregation. Sanofi-Aventis and Bristol-Myers are companies that produce Clopidogrel by the name of Clopidogrel bisulfate. Its trade name is Plavix, nonetheless in Iran it is distributed under the name of Clopidex by Exir Company. In this study we are to compare Plavix and Clopidex in terms of efficacy as well as aggregometry parameters like ADP and PRP (Platelet Rich Plasma).

**Patients and Methods:**

This is a double blind clinical trial in which we had two groups of patients suffering from Ischemic heart disease who were selected by inclusion criteria. Group A (36 patients) took Plavix (75 mg/d) and group B (36 patients) used clopidex (75 mg/d) both for 30 days. The aggregometry parameters also consisted of PRP and ADP that were run on the patients before and after the study. Finally, a comparison of aforementioned tests, quality of life, lab parameters and compliance in both groups was provided.

**Results:**

In groups A and B, the mean levels of PRP before the study were 348000 and 340000/µL respectively. The ADPs were also 73/76 and 68/07 µM that showed no significant difference (P > 0.05).The Means of ADP5 in group A before and after the study were 66.40 and 43.84 µM respectively that there was significant difference (P = 0.001). The Means of ADP5 in group B before and after the study were 58.04 and 40.16 µM respectively that there was significant difference (P < 0.001).The Means of ADP20 in group A before and after the study were 73.76 and 54.97 µM respectively which showed significant difference (P < 0.001). The Means of ADP20 in group B before and after the study were 68.07 and 52.49 µM respectively which showed significant difference (P = 0.001). Difference of ADP5 between group A and B was not significant (P = 0.495). Difference of ADP20 between group A and B was not significant (P = 0.721). The Means of PRP in group A before and after the study were 348000 and 335000/ µL respectively that there was no significant difference (P = 0.66). The Means of PRP in group B before and after the study were 340000 and 336000/ µL respectively that indicated no significant difference (P = 0.81). Difference of PRP between group A and B was not significant (P = 0.563).

**Conclusions:**

Our findings suggested that both drugs significantly lessen the ADP level; even so there was no significant difference between two groups in PRP and ADP factors.

## 1. Background

Ischemic heart disease is a condition which is characterized by an insufficient supply of blood and oxygen to myocardium. The most common cause of myocardial ischemia is atherosclerotic disease of epicardial coronary arteries that engenders reduction in myocardial blood flow, that is, inadequate perfusion of myocardium through coronary arteries. The most prevalent cause of death across industrial and developing countries is escalation of ischemic heart disease (1). Epidemiology of therapeutic regimens in acute myocardial infarction (AMI) indicates substantial increases in the use of thrombolytic therapy (2). The activation of platelets is controlled by a variety of receptors. In fact, receptors’ stimulation triggers two subsequent processes. First is the activation of internal signaling pathway and second is the binding of platelet to the adhesive protein of other thrombocytes. Several transmembrane receptors are found on the platelets, including ADP, PRP, prostaglandin, Lipid and chemokine. Adenosine receptors are responsible for transduction of ADP-induced signaling events through the binding of adenosine receptors on the platelet surface (1). Platelet inhibition seems a sure way for the prevention and treatment of ischemic heart disease and to step this way.

## 2. Objectives

Clopidogrel has markedly proved safe and efficient (3). Myocardial infraction is the most common and best-known type of heart ischemic diseases. Each year more than 32 million cases of MI are diagnosed (4). Thrombosis is the major reason for MI in patients with atherosclerosis in coronary arteries. Studies indicate that MI can shorten mean life of people, aged 60 and over, by 8-10 years (5). Although Aspirin is a chosen drug for patients with coronary intervention, adding an adenosine diphosphate (ADP) receptor antagonist (Clopidogrel) can provide greater protection from thrombotic complications (6). ADP (adenosine diphosphate) has a receptor on the platelet membrane which can be irreversibly blocked when taking an oral anti-platelet drug named clopidogrel by patient (7). Aggregation of platelets by ADP, on the grounds of a research study, can be significantly reduced via clopidogrel (P < 0.01) (8). PRP (Platelet Rich Plasma) is considered as an autologous biotherapy which is based on platelet-healing proprties. When platelets are activated, they release proteins (cytokines and growth factors) which improve regenerative process (9). Clopidogrel is produced by the name of Clopidogrel bisulfate by Sanofi-Aventis and Bristol-Myers Companies and then distributed to the market under the trade name of Palvix. In Iran this drug is produced by Exir Company under the trade name of Clopidex. As Plavix costs are very expensive in Iran, Exir, company produced it at much lower price. Because ADP and PRP are counted as the main factors of platelets’ aggregation; hence, in this study we strive to compare the aggregometery parameters of the aforemenstioned drugs looking into sample population.

## 3. Patients and Methods

This is a double blind clinical trial study that was done on patients with Ischemic Heart Disease in Baqiyatallah Hospital which is a specialized, referral hospital with 700 bed in 2012 in Tehran, Iran. The patients were selected on the basis of definite diagnosis of the disease along with qualifying for inclusion criteria and then were randomly divided into two drug groups we have totally 50 patients in each group and according to the pilot study, sample size was determined 35 patients in each group, using Alfa = 0.5, Beta = 0.9. The first group (35 patients) was given Clopidex tablets (75 mg/d) made by Exir Pharmaceutical Company and the second (35 patients) took Plavix tablets (75 mg/d) made by Sanofi-Aventis company, both for one month The rest patients were excluded from study because of non-appropriate following of drug order dose or not taking blood sampling on time. All the patients were examined by a cardiologist at the beginning of the study, one month and two months later. For all the patients, clinical and demographic data were gathered using specified questionaries. Biochemical blood tests (complete blood count, fasting blood sugar, cholesterol, triglyceride, Cratinin, Blood Urea Nitrogen) and aggregometry tests altogether were done before and after study. Besides, 10 cc of vein blood was taken from patients for detection of aggregometry parameters like ADP and PRP. Platelet aggregometry was determined through measuring OD (Optical Density) of mixed PRP after adding the agonist to aggregometer cell. Most aggregometers can be standardized and calibrated by putting patients’ PRP in the holder place of cell which indicates 0% of light transition; and if putting PPP in it, 100% of light transition occurs. Increase in light transition from 0% to 100% in recording curve or digital displayer reflects that aggregometer is reading. When the agonist is added to PRP, platelets start aggregating which results in the rise of light transition. This light is recorded and used as platelet activation standard.This study was approved by ethical committee of Baqiyatallah University and got Iranian registry of clinical trial (IRCT) code of 201011025073N1. Inclusion criteria of the patients made up of: Ischemic Heart Disease or ischemia, cooperation of patients in experiment and adherence to appropriate drug use. Exclusion criteria includes :Existence of any hemorrhagic diseases, for example peptic ulcer and intracranial hemorrhage, Existence of accompanied diseases interfering with drugs taken, history of allergy to plavix or Clopidex, any contraindication for taking Palvix or Clopidex (Thrombocytopenia, liver disorders, Neutropeni), Those of patients who needed some mechanical hemodynamic support like using balloon pump. All patients were asked to sign a consent form for participation. The Helsinki principals were considered in this study. All side effects were explained to patients and all drugs and lab tests were free of charge. Patients were free to stop taking drugs at any time during the test as well. Finally the results were compared together and statistically analyzed by SPSS software. Data were analyzed with SPSS version 17 (SPSS Inc., Chicago, Ill., USA). Continuous variables are presented as the mean ± Standard Deviation (SD) and Median, Inter Quartile Range (IQR), whereas categorical data are presented as frequency and percentages. Independent t tests or Mann-Whitney Test were used for continuous variables. In this study, the probability value of 0.05 or less (P≤0.05) was set to know the significance level.

## 4. Results

In this study, group a received plavix and group B received clopidex. The investigated aggregometery parameters in this study were PRP (Platelet rich plasma) and ADP (Adenosine diphosphate). Demographic data such as age and sex of patients in this study had no significant difference between group A and B (Table 1). In groups A and B, the mean levels of PRP before the study were 348000 and 340000/µL respectively. The ADPs were also 73/76 and 68/07 µM that showed no significant difference (P > 0.05).The Means of ADP5 in group A before and after the study were 66.40 and 43.84 µM respectively that there was significant difference (P = 0.001). The Means of ADP5 in group B before and after the study were 58.04 and 40.16 µM respectively that there was significant difference (P < 0.001).The Means of ADP20 in group A before and after the study were 73.76 and 54.97 µM respectively which showed significant difference (P < 0.001). The Means of ADP20 in group B before and after the study were 68.07 and 52.49 µM respectively which showed significant difference (P = 0.001) (Table 2). Difference of ADP5 between group A and B was not significant (P = 0.495). Difference of ADP20 between group A and B was not significant (P = 0.721) (Table 3). The Means of PRP in group A before and after the study were 348000 and 335000/ µL respectively that there was no significant difference (P = 0.66).

**Table 1.** The Comparison Demographic Parameters between two Groups

Groups	A		B		P value
	No. (%)	Mean ± SD	No. (%)	Mean ± SD	
**Sex**					0.813
Female	19 (52.8%)	-	21 (58.3%)		
Male	17 (47.2%)	-	15 (41.7%)		
**Age**	-	64.14 ± 10.92		60.94 ± 13.51	0.274

**Table 2.** The Comparison Aggrigometery Parameters between two Groups Before and After the Study ^a, b^

Group	Before		After			IQR	P value
	Mean ± SD	Median	IQR	Mean ± SD	Median		
**A**							
PRP	348000 ± 87374.12	3.62E5	108000	335000 ± 67609.66	3.40E5	118000	0.666
ADP20	73.76±18.22	3.13E5	109000	54.97 ± 16.20	3.03E5	151000	< 0.001
ADP5	66.40 ± 23.75	77.65	18.2	43.84 ± 17.92	75.00	22.3	0.001
PT Count	214000 ± 66073.28	57.30	20.85	204000 ± 52438.43	57.30	31.6	0.910
**B**							
PRP	340000 ± 88077.30	70.00	19.05	336000 ± 87315.42	63.60	36.4	0.815
ADP20	68.07 ± 15.53	49.80	31.85	52.49 ± 22.21	40.00	29.5	0.001
ADP5	58.04 ± 19.29	1.99E5	85500	40.16 ± 18.52	1.94E5	64000	< 0.001
PT Count	201000 ± 63190.72	2.04E5	87000	194000 ± 48448.50	1.91E5	41000	0.999

^a^ Abbreviations: ADP, Adenosine Diphosphate; PRP, Platelet-rich Plasma

^b^ All data are expressed as Mean ± SD or No. (%), P value base on Wilcoxon Signed Ranks Test

**Table 3.** The Comparison Difference of Aggrigometery Parameters between two Groups ^a, b^

Groups	A			B			P value
	Mean ± SD	Median	Range	Mean ± SD	Median	Range	
**PRP**	18000 ± 96153.17	4000.00	458000	9875.00 ± 102813.88	16000	458000	0.563
**ADP20**	19.81 ± 24.04	21.40	83.00	17.32 ± 23.82	21.40	82.80	0.721
**ADP5**	23.10 ± 33.02	15.50	107.30	19.00 ± 24.71	15.40	101.10	0.495
**PT Count**	13700 ± 93252.32	6000.00	365000	11500 ± 90876.73	3000.00	365000	0.995

^a^ Abbreviations: ADP, Adenosine Diphosphate; PRP, Platelet-rich Plasma

^b^ All data are expressed as Mean ± SD, P value base on Mann-Whitney test

The Means of PRP in group B before and after the study were 340000 and 336000/ µL respectively that indicated no significant difference (P = 0.81). Difference of PRP between group A and B was not significant (P = 0.563) (Table 3). The platelet count in group A showed no significant differences before and after the study (P = 0.910) and in group B the situation was the same either (P = 0.999). The differences between two groups was also the same (P = 0.994) (Table 3). The quality of life based on VAS standard, before and after study in group A and B had a significant difference (P < 0.05), but the difference between two groups was non-significant (P > 0.05). There were no significant differences between two groups with regard to the laboratory parameters (PT, PTT, FBS, BUN, Cr, TG, and Cho) (P > 0.05). There was not seen any significant difference between two groups as to compliance and adverse drug reaction, (P > 0.05).

## 5. Discussion

Ischemic Heart disease is a condition characterized by inadequate supply of blood and oxygen to myocardium. Epidemiology of therapeutic regimens for acute myocardial infarction (AMI) indicates considerable increases in the use of thrombolytic therapy (2). The activation of platelets is controlled by a variety of receptors. Several transmembrane receptors are found on platelets, namely ADP and PRP. Many of clinical trials have shown the long-term benefits of antiplatelet drugs for decrease in the risk of thrombotic accidents. Clopidogrel is a thienopyridine derivative which prevents platelets from adhering together by direct inhibition of adenosine diphosphate, that is, the factor behind platelets’ aggregation (10).

Our findings showed that PRP parameter in group A and B had no significant difference before and after the study (P = 0.666) and the difference of PRP between groups A and B was not significant. The ADP parameter with concentrations of 5 and 20, was significantly reduced in both groups after the study, Even so there was no significant difference between groups A and B. The ADP in concentration of 5, was significantly reduced in group A, after the study (P = 0.001) and in group B the situation was the same either (P < 0.001), but the differences between groups A and B were non-significant (P = 0.495) (Table 3). The ADP in both concentration (5 and 20) in group A and B showed no significant difference.

In a randomized double blind clinical trial that was performed to evaluate the efficacy and safety of Clopidogrel and aspirin in decreasing the risk of ischemic stroke and MI, it turned out that the long term use of Clopidogrel in patients with atherosclerotic vascular disease is more effective than aspirin. However there was no significant difference in safety (10).

In a research study, 197 patients who had undergone coronary artery bypass graft (CABG) were devided into two groups. 102 patients took Clopidogrel (75 mg/d) and 95 patients used Clopidogrel (75 mg/d) plus Aspirin (100 mg/d). There was no significant difference in graft patency between two groups (P > 0.05) (11).

In another study, 70 randomly chosen patients with ischemic stroke were divided into two drug groups. One group received aspirin and the other received aspirin plus Clopidogrel. Platelets studies were performed on both groups. Treatment with Clopidogrel plus aspirin had a distinctively better platelet inhibition activity compared to aspirin itself in patients after ischemic stroke (12). To find out the spread of aspirin resistance, a clinical study on patients who had acute coronary syndrome was done. The function of platelets was analyzed through platelet function analyzer (PFA) on 100 patients in terms of collagen or ADP (Col/ADP) and collagen or epimephrine (Col/Epi). The results showed that 19% (n = 20) of the patients put up resistance to aspirin (13).

In another study Joanna Fong et al. evaluated the rate of biochemical response of aspirin and clopidogrel. The results suggested that biochemical nonresponse to antiplatelet drugs may occur in ischemic heart disease and different factors can affect this response (8). Milionis et al. compared the effect of aspirin and clopidogrel on patients who had ischemic heart stroke for the first time. They were given aspirin (n = 880) and clopidogrel (n = 348). In the initial 6 months of treatment through these drugs, the distinction of survival was obvious; it was 93.8% for aspirin and 97% for clopidogrel. In addition, the collection of cardiovascular accidents was lower in patients treated with clopidogrel (n = 60, 17.2%) vs. those with aspirin (n = 249, 28.3%) (P < 0.0001) (14). In a research study, a novel dynamic layer-by-layer (d-LbL) biointerface on a nano-scale was developed which functioned as an anti-coagulation surface. It was utilized as a biologically-active substrate for platelet adhesion and aggregation. Totally, PRP + ADP was more effective at increasing platelet aggregation (15). Boris Shenkman et al. studied the Unresponsiveness to antiplatelet drugs like clopidogrel and aspirin in acute coronary syndrome patients (ACS). In a laboratory study 404 patients were selected. On days 1 and 4 the first group (n = 114) was evaluated with PA, and then a patient with a decrease less than 10% in ADP induced after treatment with clopidogrel, was called a non-responding (NR) to clopidogrel. This value is correlated with the aggregation of ADP-induced more than 70%. Besides, a patient who had a value for AA-induced aggregation more than 60% was described a non-responding (NR) to aspirin. On day 4 the second group was evaluated by both previous methods and the results were respectively: less than 2.8% and more than 3.4% for clopidogrel and aspirin NR. The NR incidence for clopidogrel and aspirin was 22% and 27% respectively (16). In another study, the clopidogrel or aspirin resistance of patients with acute coronary syndrome (ACS) was ascribed to metabolic syndromes (MS). The factors using for this aim were platelet-rich plasma (PRP), collagen and adenosine diphosphate (ADP). High levels of resistance to antiplatelet drugs were determined in MS patients. ACS patients without MS also showed this resistance to a significant degree (17). In 2009 Massie BM et al. carried out the treatment applying warfarin and clopidogrel or aspirin during a period of 30 months. 1587 patients who had heart failure within the past 3 months were enrolled. Final results indicated that warfarin is not superior to aspirin and clopidogrel (18). Our findings suggested that both drugs significantly lessen the ADP level; even so there was no significant difference between two groups in PRP and ADP factors. This research study suggests that aggregometry parameters indicate no significant differences in patients who receive Plavix or clopidex.

## References

[1] Longo DL, Fauci AS, Kasper DL, Hauser SL, Jameson JL, Loscalzo J (1998). Harrisons Principles of internal Medicine..

[2] Tavazzi L (1999). Clinical epidemiology of acute myocardial infarction.. Am Heart J..

[3] Galla JM, Lincoff AM (2007). The role of clopidogrel in the management of ischemic heart disease.. Curr Opin Cardiol..

[4] Peeters A, Mamun AA, Willekens F, Bonneux L (2002). A cardiovascular life history. A life course analysis of the original Framingham Heart Study cohort.. Eur Heart J..

[5] Kosuge M, Kimura K, Ishikawa T, Ebina T, Hibi K, Tsukahara K (2006). Differences between men and women in terms of clinical features of ST-segment elevation acute myocardial infarction.. Circ J..

[6] Leon MB, Baim DS, Popma JJ, Gordon PC, Cutlip DE, Ho KK (1998). A clinical trial comparing three antithrombotic-drug regimens after coronary-artery stenting. Stent Anticoagulation Restenosis Study Investigators.. N Engl J Med..

[7] Mehta SR, Yusuf S, Peters RJ, Bertrand ME, Lewis BS, Natarajan MK (2001). Effects of pretreatment with clopidogrel and aspirin followed by long-term therapy in patients undergoing percutaneous coronary intervention: the PCI-CURE study.. Lancet..

[8] Fong J, Cheng-Ching E, Hussain MS, Katzan I, Gupta R (2011). Predictors of biochemical aspirin and clopidogrel resistance in patients with ischemic stroke.. J Stroke Cerebrovasc Dis..

[9] Bausset O, Giraudo L, Veran J, Magalon J, Coudreuse JM, Magalon G (2012). Formulation and storage of platelet-rich plasma homemade product.. Biores Open Access..

[10] Caprie Steering Committee. (1996). A randomised, blinded, trial of clopidogrel versus aspirin in patients at risk of ischaemic events (CAPRIE). CAPRIE Steering Committee.. Lancet..

[11] Gao C, Ren C, Li D, Li L (2009). Clopidogrel and aspirin versus clopidogrel alone on graft patency after coronary artery bypass grafting.. Ann Thorac Surg..

[12] Serebruany VL, Malinin AI, Ziai W, Pokov AN, Bhatt DL, Alberts MJ (2005). Effects of clopidogrel and aspirin in combination versus aspirin alone on platelet activation and major receptor expression in patients after recent ischemic stroke: for the Plavix Use for Treatment of Stroke (PLUTO-Stroke) trial.. Stroke..

[13] Pamukcu B, Oflaz H, Oncul A, Umman B, Mercanoglu F, Ozcan M (2006). The role of aspirin resistance on outcome in patients with acute coronary syndrome and the effect of clopidogrel therapy in the prevention of major cardiovascular events.. J Thromb Thrombolysis..

[14] Milionis HJ, Gerotziafas G, Kostapanos MS, Vemmou A, Zis P, Spengos K (2011). Clopidogrel vs. aspirin treatment on admission improves 5-year survival after a first-ever acute ischemic stroke. data from the Athens Stroke Outcome Project.. Arch Med Res..

[15] Watson MG, Lopez JM, Paun M, Jones SA (2013). A novel dynamic layer-by-layer assembled nano-scale biointerface: functionality tests with platelet adhesion and aggregate morphology influenced by adenosine diphosphate.. J Thromb Thrombolysis..

[16] Shenkman B, Matetzky S, Fefer P, Hod H, Einav Y, Lubetsky A (2008). Variable responsiveness to clopidogrel and aspirin among patients with acute coronary syndrome as assessed by platelet function tests.. Thromb Res..

[17] Paul R, Banerjee AK, Guha S, Chaudhuri U, Ghosh S, Mondal J (2013). Study of platelet aggregation in acute coronary syndrome with special reference to metabolic syndrome.. Int J Appl Basic Med Res..

[18] Massie BM, Collins JF, Ammon SE, Armstrong PW, Cleland JG, Ezekowitz M (2009). Randomized trial of warfarin, aspirin, and clopidogrel in patients with chronic heart failure: the Warfarin and Antiplatelet Therapy in Chronic Heart Failure (WATCH) trial.. Circulation..

